# Racial/Ethnic Differences in Associations Between Neighborhood Social Cohesion and Meeting Physical Activity Guidelines, United States, 2013–2014

**DOI:** 10.5888/pcd13.160261

**Published:** 2016-12-08

**Authors:** Stella S. Yi, Chau Trinh-Shevrin, Irene H. Yen, Simona C. Kwon

**Affiliations:** 1 New York University School of Medicine, New York, New York; 2 University of California, San Francisco, San Francisco, California.

## Abstract

**Introduction:**

Neighborhood factors are increasingly recognized as determinants of health. Neighborhood social cohesion may be associated with physical activity, but previous studies examined data aggregated across racial/ethnic groups. We assessed whether neighborhood social cohesion was associated with physical activity in a nationally representative data set and explored the role of race/ethnicity.

**Methods:**

We combined National Health Interview Survey data from 2013 and 2014 (n = 64,754) and constructed a neighborhood social cohesion score by summing responses to 4 questions. The outcome of meeting aerobic physical activity guidelines was defined as 150 or more minutes per week of moderate activity or 75 or more minutes of vigorous activity. Multivariable models regressing physical activity on neighborhood social cohesion were adjusted for demographic factors; interaction analyses assessed effect modification by race/ethnicity.

**Results:**

In adjusted analyses, a 1-unit increase in the neighborhood social cohesion score was associated with higher odds of meeting physical activity guidelines (odds ratio [OR], 1.04; 95% confidence interval [CI], 1.03–1.05). Neighborhood social cohesion and physical activity were associated among non-Hispanic white adults (OR, 1.30; 95% CI, 1.20–1.42) and Hispanic adults (OR, 1.18; 95% CI, 1.03–1.34]) but not among non-Hispanic black or Asian American adults (Chinese, Filipino, and Asian Indians).

**Conclusion:**

Neighborhood social cohesion was associated with meeting physical activity guidelines in a nationally representative sample; this association may be most meaningful for non-Hispanic white and Hispanic populations. Additional studies are needed to identify neighborhood factors that help non-Hispanic black and Asian Americans to meet physical activity guidelines.

## Introduction

Physical activity is a beneficial health behavior, associated with reduced risk of several health conditions including obesity, cancer, and other chronic diseases (1–4). According to national data published in 2014, only 36% of adults were aware of the 2008 Physical Activity Guidelines for Americans (5) and only 52% of adults met the aerobic guidelines (6). Racial/ethnic disparities exist for physical activity: racial/ethnic minority populations are less likely to be physically active than the white population (7,8), and Asian Americans have the lowest activity levels (9,10).

Neighborhood factors are increasingly recognized as determinants of health and of health behaviors, including physical activity (11–16). Neighborhood social cohesion, a construct of the social environment, is the perceived degree of connectedness between and among neighbors and their willingness to intervene for the common good (11). Neighborhood social cohesion may influence physical activity behaviors through greater social support or communication, reinforcement of social norms (positive or negative) for exercise, or greater access to parks or green spaces (11,17).

Neighborhood social cohesion has been associated with meeting physical activity guidelines and regular physical activity (18). However, the findings of analyses disaggregated by racial/ethnic group are mixed. Analyses of California Health Interview Survey data showed that neighborhood social cohesion was associated with walking for physical activity among white and Hispanic adults but not among black and Asian American adults (19) and among older Chinese adults but not Filipino, Japanese, Korean, or Vietnamese adults (20).

The objectives of this analysis were to 1) characterize factors associated with higher levels of neighborhood social cohesion, 2) assess the association between neighborhood social cohesion and aerobic physical activity in a large, nationally representative data set, and 3) explore the role of race/ethnicity as a potential confounder or effect modifier in the association between neighborhood social cohesion and physical activity. We hypothesized that neighborhood social cohesion and aerobic physical activity are positively associated and that this association would be modified by race/ethnicity.

## Methods

We combined data from the National Health Interview Survey (NHIS) 2013 and 2014 survey waves (n = 71,254 adults). NHIS is an annual cross-sectional health survey conducted among the US population in English and Spanish continuously throughout the year (21,22). Data are self-reported and collected by trained interviewers in the US Census Bureau; interviewers use computer-assisted personal interviewing, a data collection method in which an interviewer meets with a respondent face-to-face to ask questions and enter answers into a laptop computer.

### Definitions of key variables

The NHIS in 2013 and 2014 included 4 questions on neighborhood social cohesion, modified from an original scale of 5 questions developed by the Project on Human Development in Chicago Neighborhoods Community Survey (23). Participants rated agreement or disagreement on a 4-point scale (1, definitely agree; 2, somewhat agree; 3, somewhat disagree; and 4, definitely disagree) with the following 4 statements: 1) People in this neighborhood help each other out; 2) There are people I can count on in this neighborhood; 3) People in this neighborhood can be trusted; and 4) This is a close-knit neighborhood. Participant responses were then reverse coded (eg, definitely agree was assigned a value of 4 instead of 1); thus a higher score equated higher neighborhood social cohesion. A neighborhood social cohesion score was constructed by summing the responses to the questions, with a possible range of scores from 4 to 16. Neighborhood social cohesion was treated as both a continuous variable (ie, difference associated with 1-unit change in score) and as a binary variable, categorized as at or above the median score or below the median score.

Using a series of 8 questions, the NHIS measured leisure-time physical activity. Vigorous activity was assessed by asking 2 questions, starting with, “How often do you do vigorous leisure-time physical activities for at least 10 minutes that cause heavy sweating or large increases in breathing or heart rate?” The next question asked about frequency (per day, week, month, and year). Participants also reported on how long they performed these vigorous activities. Similarly, moderate activity was assessed by starting with the following question: “How often do you do light or moderate leisure-time physical activities for at least 10 minutes that cause only light sweating or a slight to moderate increase in breathing or heart rate?” Frequency and how long they performed these moderate activities were assessed in subsequent questions. Data on physical activity were cleaned and recoded as per NHIS protocol for leisure-time physical activity (24). The primary outcome was defined as meeting guidelines on aerobic physical activity (≥150 minutes per week of moderate or ≥75 minutes per week of vigorous physical activity) (25). As per protocol for defining moderate-equivalent minutes, minutes per week of vigorous physical activity were multiplied by 2 (24). The secondary outcome was total number of moderate or moderate-equivalent minutes per week of aerobic physical activity, modeled as a continuous variable.

Other covariates were age (18–44, 45–64, or ≥65 y), racial/ethnic group (non-Hispanic white, non-Hispanic black, Hispanic, non-Hispanic Chinese, non-Hispanic Filipino, or non-Hispanic Asian Indian), sex, education (data restricted to those aged ≥25 y [<high school diploma, high school diploma or equivalent, some college, college graduate, or graduate degree]), annual income (<$20,000, $20,000 to <$45,000, $45,000 to <$75,000, or ≥$75,000), nativity (born in United States or foreign born) and English language proficiency (speaks very well or not very well). Length of time living in one’s neighborhood was assessed with the question, “About how long have you lived in your present neighborhood?”; response choices were less than 1 year, 1 to 3 years, 4 to 10 years, 11 to 20 years, and more than 20 years.

### Statistical analysis

Of the 71,254 adults in the 2 years of the NHIS, 5,663 respondents were missing data on neighborhood social cohesion variables, and 1,276 respondents were missing data on the physical activity variables needed to calculate whether they met physical activity guidelines; these respondents were not included in the analysis. The final analytic sample size was 64,754 (439 respondents were missing both neighborhood social cohesion and physical activity).

Data were weighted to be representative of the noninstitutionalized adult population in the United States. Demographic and other characteristics were assessed overall and then stratified by median neighborhood social cohesion score (at or above median score or below median score). Differences between those at or above the median score and those below the median score were assessed by using χ^2^ tests. We also estimated mean neighborhood social cohesion score. To characterize factors associated with higher levels of neighborhood social cohesion, all covariates (age group, race/ethnicity, sex, education, annual income, nativity, English language proficiency, and length of time in neighborhood) were included in a multivariable regression model.

We ran multivariable models regressing physical activity outcomes on neighborhood cohesion; we used logistic regression models for the binary outcome of meeting physical activity guidelines, and linear regression models for the continuous outcome of moderate or moderate-equivalent minutes per week of physical activity. Both models were run as crude models, then adjusted for race/ethnicity, and then adjusted for age, race/ethnicity, sex, education, annual income, nativity, English language proficiency, and length of time living in the neighborhood. Education and annual income were moderately correlated (ρ = 0.38, *P* < .001), so we ran models that excluded one or the other. Results did not change substantially in either model; thus, our final models adjusted for both variables. To assess effect modification by race/ethnicity, we ran a logistic regression model that included an interaction term between race/ethnicity and binary neighborhood cohesion. All analyses were conducted using complex survey weighting techniques, and data were analyzed using Stata version 12.1 (StataCorp LP) and Sudaan version 11.0.1 (RTI International).

## Results

The greatest portion (46.7%) of the overall sample was aged 18 to 44; 67% were non-Hispanic white, 51.7% were women, and 44.3% had a college or graduate degree (Table 1). The median neighborhood social cohesion score was 12.0. Neighborhood social cohesion scores at or above the median were associated with all demographic characteristics examined: older age, non-Hispanic white race/ethnicity, female sex, having a college degree and higher income, being born in the United States, English language proficiency, and having lived in the neighborhood longer. Neighborhood social cohesion scores at or above the median were also associated with a slightly higher prevalence of meeting aerobic physical activity guidelines (51.5% for scores ≥median vs 45.6% for scores <median; *P* = .001) and with a greater number of moderate or moderate-equivalent minutes per week of physical activity (371.0 min/week for scores ≥median vs 319.3 min/week for scores <median, *P* = .001). The overall mean neighborhood social cohesion score was 12.4. After adjustment for each other, the following covariates remained associated with neighborhood social cohesion: being aged 65 or older (0.41 score units higher than the score for those aged 18–44; *P* < .001); being non-Hispanic black (1.00 score units lower than the score for non-Hispanic whites; *P* < .001) or Hispanic (0.72 score units lower than the score for non-Hispanic whites; *P* < .001); having higher education levels (0.37 score units higher for some college, 0.48 score units higher for college graduate, and 0.70 score units higher for graduate degree, all compared with less than a high school diploma, *P* < .001 for all); having higher income levels (0.38 score units higher for those with incomes of $45,000 to <$75,000 and 0.71 score units higher for those with incomes of ≥$75,000, compared with incomes of <$20,000; *P* < .001 for both); and having lived longer in the neighborhood (0.70 score units higher for those in the neighborhood for 4–10 y, 1.08 score units higher for those in the neighborhood for 11–20 y, and 1.30 score units higher for those in the neighborhood for >20 y, compared with those in the neighborhood for <1 y; *P* < .001 for all). The correlation between neighborhood social cohesion and length of time living in the neighborhood was weak but significant (ρ = 0.17, *P* < .001).

**Table 1:** Demographic Characteristics of US Adults, Overall and by Neighborhood Social Cohesion^a^, National Health Interview Survey, 2013–2014^b^

Characteristic	Overall		Neighborhood Social Cohesion Score^c^				*P* Value^d^
			At or Above Median		Below Median		
	Unweighted No.	Weighted %^b^	Unweighted No.	Weighted %^b^	Unweighted No.	Weighted %^b^	
**Overall**	64,754	100	42,972	—	21,782	—	—
**Age group, y**							
18–44	27,980	46.7	16,705	43.0	11,275	54.4	.001
45–64	21,830	34.8	14,867	35.9	6,963	32.5	
≥65	14,944	18.5	3,544	21.1	3,544	13.0	
**Race/ethnicity**							
Non-Hispanic white	40,225	67.0	28,788	71.4	11,437	57.7	.001
Non-Hispanic black	9,088	11.6	5,255	9.9	3,833	15.0	
Hispanic	10,808	15.0	5,912	12.3	4,896	20.7	
Non-Hispanic Chinese	778	1.1	496	1.1	282	1.1	
Non-Hispanic Filipino	841	1.2	587	1.2	254	1.0	
Non-Hispanic Asian Indian	717	1.2	536	1.4	181	0.8	
**Sex**							
Male	28,957	48.3	19,499	48.8	9,458	47.3	.004
Female	35,797	51.7	23,473	51.2	12,324	52.7	
**Education^e^**							
<High school diploma	9,029	13.2	5,442	11.6	3,587	16.9	.001
Grade 12 or GED	15,006	25.3	9,899	24.3	5,107	27.3	
Some college	10,262	17.3	6,817	17.1	3,445	17.6	
College graduate	17,667	32.2	12,471	33.5	5,196	29.2	
Graduate degree	6,556	12.1	4,930	13.4	1,626	9.0	
**Annual income, $**							
<20,000	10,581	29.0	6,319	26.4	4,262	34.3	.001
20,000 to <45,000	12,316	33.5	7,743	32.0	4,573	36.4	
45,000 to <75,000	7,156	21.2	5,022	22.5	2,134	18.5	
≥75,000	4,918	16.4	3,788	19.0	1,130	10.8	
**Nativity**							
Born in United States	52,885	82.1	35,940	83.9	16,945	78.4	.001
Foreign born	11,852	17.9	7,021	16.1	4,831	21.6	
**How well English is spoken**							
Very well	43,463	88.7	29,533	90.4	13,930	85.0	.001
Not very well	6,273	11.3	3,593	9.6	2,680	15.0	
**Length of time living in neighborhood, y**							
<1	8,227	11.7	4,685	10.1	3,542	15.2	.001
1–3	13,567	19.9	7,933	17.6	5,634	25.0	
4–10	17,015	27.0	11,259	23.9	5,756	27.1	
11–20	11,517	19.5	8,175	20.9	3,342	16.7	
>20	14,369	21.8	10,886	24.5	3,483	16.0	
**Meeting aerobic physical activity guidelines^f^**	31,107	49.6	21,345	51.5	9,762	45.6	.001
**Moderate or moderate-equivalent minutes per week of physical activity, mean**	64,290	354.3	42,646	371.0	21,644	319.3	.001

Abbreviations: GED, general educational development.

a The perceived degree of connectedness between and among neighbors and their willingness to intervene for the common good (11).

b All estimates are weighted to be representative of the US adult noninstitutionalized population.

c Constructed by summing the responses to 4 questions: 1) People in this neighborhood help each other out; 2) There are people I can count on in this neighborhood; 3) People in this neighborhood can be trusted; and 4) This is a close-knit neighborhood. Scores could range from 4 to 16, with higher scores indicating greater agreement with statements. The median score was 12.0.

d Determined by using *t* tests for continuous variables and *t* tests for proportions for categorical variables.

e Data on education were restricted to respondents aged ≥25 y.

f Defined as 150 or more minutes per week of moderate activity or 75 or more minutes of vigorous activity (25).

After adjustment for all covariates, the odds of meeting aerobic physical activity guidelines associated with a 1-unit increase in the neighborhood social cohesion score were 1.04 (95% confidence interval [CI], 1.03–1.05; *P* < .001), whereas the odds of meeting aerobic physical activity guidelines were 1.22 (95% CI, 1.13–1.32; *P* < .001) for a neighborhood social cohesion score at or above the median compared with a score below the median (Table 2). Results did not change substantially from the crude model when we adjusted for race/ethnicity. A 1-unit increase in the neighborhood social cohesion score was associated with 6.9 minutes (95% CI, 3.5–10.4 min; *P* < .001) more of moderate or moderate-equivalent minutes per week of physical activity after adjustment. Similarly, a neighborhood social cohesion score at or above the median was associated with 45.3 minutes (95% CI, 22.1–68.6 min; *P* < .001) more of moderate or moderate-equivalent physical activity minutes per week compared with a social cohesion score below the median. In interaction analyses, the overall interaction term was significant (*P* = .003). After adjustment, consistent associations between neighborhood social cohesion and aerobic physical activity remained among non-Hispanic whites only; those who had neighborhood social cohesion scores at or above the median had a higher odds of meeting aerobic physical activity guidelines (OR = 1.30; 95% CI, 1.20–1.42; *P* < .001; Table 3) and had more moderate or moderate-equivalent minutes per week of physical activity (58.1 minutes; 95% CI, 30.4–85.8 min; *P* < .001) than those with scores below the median. For Hispanic respondents, we found greater odds of meeting aerobic physical activity guidelines among those reporting neighborhood social cohesion scores at or above the median than among those reporting neighborhood social cohesion scores below the median (OR = 1.18; 95% CI, 1.03–1.34, *P* = .01).

**Table 2:** Multivariable Regression Models, Neighborhood Social Cohesion^a^ Score^b^ and Physical Activity Outcomes, National Health Interview Survey, 2013–2014

Variable	Crude	Adjusted for Race/Ethnicity	Adjusted for All Covariates^c^
**Meeting aerobic physical activity guidelines^d^, OR (95% CI) [*P* value^e^]**			
Neighborhood social cohesion score, per 1-unit change	1.04 (1.03–1.05) [<.001]	1.04 (1.03–1.05) [<.001]	1.04 (1.03–1.05) [<.001]
Neighborhood social cohesion score at or above vs below median	1.22 (1.20–1.33) [<.001]	1.22 (1.16–1.28) [<.001]	1.22 (1.13–1.32) [<.001]
**Moderate-equivalent minutes per week of physical activity, β coefficient (95% CI) [*P* value^e^]**			
Neighborhood social cohesion score, per 1-unit change	7.8 (5.5–10.1) [<.001]	7.0 (4.7–9.4) [<.001]	6.9 (3.5–10.4) [<.001]
Neighborhood social cohesion score at or above vs below median	51.7 (36.5–66.8) [<.001]	47.6 (32.5–62.7) [<.001]	45.3 (22.1–68.6) [<.001]

Abbreviations: CI, confidence interval; OR, odds ratio.

a The perceived degree of connectedness between and among neighbors and their willingness to intervene for the common good (11).

b Constructed by summing the responses to 4 questions: 1) People in this neighborhood help each other out; 2) There are people I can count on in this neighborhood; 3) People in this neighborhood can be trusted; and 4) This is a close-knit neighborhood. Scores could range from 4 to 16, with higher scores indicating greater agreement with statements. The median score was 12.0.

c All covariates are age, sex, race/ethnicity, education, annual income, nativity (US born or not), English language proficiency, and length of time in neighborhood.

d Defined as 150 or more minutes per week of moderate activity or 75 or more minutes of vigorous activity (25).

e Determined by using multivariable logistic (categorical outcome) or linear (continuous outcome) regression.

**Table 3:** Effect Modification of Neighborhood Social Cohesion^a^ on Physical Activity Outcomes, by Race/Ethnicity, National Health Interview Survey, 2013–2014

Neighborhood Social Cohesion Score^b^	Meeting Aerobic Physical Activity Guidelines^c^, OR (95% CI)	*P* Value^d^	Moderate-Equivalent Physical Activity^e^, Min/Week, β Coefficient (95% CI)	*P* Value^d^
**Overall**				
Below median	1 [Reference]	<.001	1 [Reference]	<.001
At or above median	1.22 (1.13 to 1.32)		45.3 (22.1 to 68.6)	
**By race/ethnicity^f^**				
Non-Hispanic white	1.30 (1.20 to 1.42)	<.001	58.1 (30.4 to 85.8)	<.001
Non-Hispanic black	0.97 (0.84 to 1.10)	.54	34.0 (−17.7 to 85.7)	.24
Hispanic	1.18 (1.03 to 1.34)	.01	5.5 (−32.3 to 43.3)	.62
Non-Hispanic Chinese	0.80 (0.56 to 1.13)	.23	−35.4 (−101.4 to 30.6)	.45
Non-Hispanic Filipino	0.88 (0.63 to 1.24)	.52	0.1 (−65.5 to 65.7)	.80
Non-Hispanic Asian Indian	1.17 (0.83 to 1.66)	.27	−12.0 (−88.6 to 64.6)	.99

Abbreviations: Abbreviations: CI, confidence interval; OR, odds ratio.

a The perceived degree of connectedness between and among neighbors and their willingness to intervene for the common good (11).

b Constructed by summing the responses to 4 questions: 1) People in this neighborhood help each other out; 2) There are people I can count on in this neighborhood; 3) People in this neighborhood can be trusted; and 4) This is a close-knit neighborhood. Scores could range from 4 to 16, with higher scores indicating greater agreement with statements. The median score was 12.0.

c Defined as 150 or more minutes per week of moderate activity or 75 or more minutes of vigorous activity (25).

d *P* values determined using multivariable logistic (categorical outcome) and linear (continuous outcome) regression.

e For defining moderate-equivalent minutes, minutes per week of vigorous physical activity were multiplied by 2 (24).

f In this analysis, the reference group for each OR and β coefficient is the group reporting a neighborhood social cohesion score below the median. For example, non-Hispanic whites reporting a neighborhood social cohesion score at or above the median were 1.30 times as likely to report meeting aerobic physical activity guidelines than non-Hispanic whites reporting a neighborhood social cohesion score below the median.

## Discussion

In a nationally representative sample of noninstitutionalized adults in the United States, higher levels of neighborhood social cohesion were associated with higher odds of meeting aerobic physical activity guidelines and more moderate or moderate-equivalent minutes of physical activity per week. The effect estimate observed in our study for the odds of meeting aerobic physical activity guidelines (OR = 1.04; 95% CI, 1.03–1.05) was similar in magnitude to a previous study that examined the same association in a multiethnic sample of US adults (OR = 1.03; 95% CI, 1.01–1.05) (18). Our study expands on this previous study by using interaction analyses to assess data for various racial/ethnic groups. In our study, the association may have been driven primarily by non-Hispanic white adults and to a lesser degree, Hispanic adults. A similar result was observed in an analysis of data representative of the adult population in California: higher levels of neighborhood social cohesion were associated overall with more walking (OR, 1.09; 95% CI, 1.04–1.14), but by racial/ethnic group, only among non-Hispanic white and Hispanic adults (19).

Neighborhood social cohesion in our study was moderately high, with a mean value of 12.4 of a maximum value of 16. The factors associated with higher levels of neighborhood social cohesion included older age, being non-Hispanic white or a college graduate, having a higher annual income, and living in the neighborhood for more than 20 years. These results are unsurprising but offer insight into the adult populations in the United States who are likely to perceive and report higher levels of neighborhood social cohesion. Similarly, one can imagine that people reporting higher levels of neighborhood social cohesion may also be those who have higher levels of health literacy, better access to safe facilities for exercise, and more time and means to access those facilities. Future analyses examining the potential mediating effects of these factors on the association between neighborhood social cohesion and activity behaviors are warranted.

Although we did not find any associations between neighborhood social cohesion and physical activity among non-Hispanic Chinese adults, non-Hispanic Filipino adults, or non-Hispanic Asian Indian adults, other studies using regional samples and in-language recruitment found different results. For example, an analysis using data on adults aged 55 or older from the California Health Interview Survey found that higher levels of neighborhood social cohesion were associated with lower odds of not walking for leisure or transport among Chinese Americans (OR = 0.39; 95% CI, 0.17–0.89) but not among Filipino, Korean, or Vietnamese Americans (20). In an analysis using data from a cross-sectional health assessment of foreign-born Chinese American immigrants in New York City (n = 1,772), higher levels of neighborhood social cohesion were associated with higher odds of meeting physical activity guidelines and with less sitting time (unpublished data, S.S.Y. et al, July 2016). Because the NHIS is conducted only in English and Spanish, it may recruit a nonrepresentative sample of immigrants (26,27) (ie, who are highly acculturated or US born, have high incomes, or do not live in urban areas), and the differences in samples may explain discrepancies in observations across these studies. The analysis of Chinese American immigrants in New York City in particular offers evidence that health literacy may be less of a factor in the association between neighborhood social cohesion and activity behaviors, because the cohort recruited had fairly low socioeconomic and acculturation status. Instead, perhaps ready access to safe places to exercise (eg, green space and gyms in suburban areas, high walkability in urban areas) may be more of a mediating factor.

The strengths of this study are large sample sizes, even for subgroups, and that results are applicable broadly to the adult population in the United States. Although physical activity was not directly measured, the use of a series of questions and self-reported data are considered valid and acceptable methods for surveying the physical activity behaviors of large samples of people (28). The limitations of this analysis are that data were collected by self-report, making responses prone to social desirability bias. The dichotomous variable of meeting aerobic physical activity guidelines may be more meaningful than the continuous values of physical activity. The ideal approach would be to use detailed biometric data obtained through direct observation, time diaries, and metabolic measures; however, at the population level, this approach is cost prohibitive (29). Thus, for assessing physical activity in large populations, self-reported responses on questionnaires, although not perfect, is a feasible method (28). The results of this study are generalizable only to the English-speaking and Spanish-speaking adult populations in the United States. Lastly, because this study was cross-sectional, the directionality of cause and effect of the association between neighborhood social cohesion and physical activity cannot be established.

Although social factors such as neighborhood social cohesion have become salient in public health, the applicability of these factors in the context of race/ethnicity or cultural nuance needs to be carefully examined. Future analyses should focus on racial/ethnic groups and subgroups (eg, Mexican vs Puerto Rican, Chinese vs Vietnamese) to fully characterize the effects of these broad social determinants of health. In addition, studies are needed to determine factors that predict physical activity behaviors for non-Hispanic black and Asian American populations, acknowledging that “one size does not fit all” in the prevention of chronic disease.
